# Mechanism for the Intercalation of Aniline Cations into the Interlayers of Graphite

**DOI:** 10.3390/nano12142486

**Published:** 2022-07-20

**Authors:** Yifan Guo, Ying Li, Wei Wei, Junhua Su, Jinyang Li, Yanlei Shang, Yong Wang, Xiaoling Xu, David Hui, Zuowan Zhou

**Affiliations:** 1 Key Laboratory of Advanced Technologies of Materials (Ministry of Education), School of Materials Science and Engineering, Southwest Jiaotong University, Chengdu 610031, China; yfguo@swjtu.edu.cn (Y.G.); tzweir@swjtu.edu.cn (W.W.); sjh@my.swjtu.edu.cn (J.S.); ylshang@my.swjtu.edu.cn (Y.S.); yongwang1976@163.com (Y.W.); bihan_2001@163.com (X.X.); 2 Research Institute of Frontier Science, Southwest Jiaotong University, Chengdu 610031, China; 3 Yibin Research Institute, Southwest Jiaotong University, Yibin 644000, China; 4 School of Mechanical Engineering, Chengdu University, Chengdu 610106, China; liying@cdu.edu.cn; 5 Department of Mechanical Engineering, University of New Orleans, New Orleans, LA 70148, USA; dhui@uno.edu

**Keywords:** intercalation, polymerization, aniline cations, graphite

## Abstract

The dynamic behaviors of aniline cation (ANI^+^) intercalating into graphite interlayers are systematically studied by experimental studies and multiscale simulations. The in situ intercalation polymerization designed by response surface methods implies the importance of ultrasonication for achieving the intercalation of ANI^+^. Molecular dynamics and quantum chemical simulations prove the adsorption of ANI^+^ onto graphite surfaces by cation–π electrostatic interactions, weakening the π–π interactions between graphene layers. The ultrasonication that follows breaks the hydrated ANI^+^ clusters into individual ANI^+^. Thus, the released positive charges of these dissociative cations and reduced steric hindrance significantly improve their intercalation ability. With the initial kinetic energy provided by ultrasonic field, the activated ANI^+^ are able to intercalate into the interlayer of graphite. This work demonstrates the intercalation behaviors of ANI^+^, which provides an opportunity for investigations regarding organic-molecule-intercalated graphite compounds.

## 1. Introduction

Due to its natural layer-stacking structure, graphite has long been intercalated for applications in superconductivity [1], catalysts [2], hydrogen storage [3], and ion batteries [4]. Hundreds of graphite intercalation compounds have been synthesized in the past two hundred years, but the intercalants are mostly strong oxidants or reductants involving alkali metal, alkaline-earth metal, halogen, and strong oxidizing acids [5,6,7,8]. Different from montmorillonite [9,10] and layer-stacked MXenes [11,12], graphite possesses a reduced stacking distance of 0.34 nm and a clean interlayer space. Therefore, the intercalation of organic molecules into graphite interlayers is difficult. In recent years, some organic molecules have achieved their intercalation with the assistance of an external electric field [13,14,15], or by cointercalating with coordination bonded alkali metals [16,17]. These intercalating molecules exhibit a controllable orientation in the interlayers [16,18] and significantly influence the properties of intercalated graphite [14,17,19,20]. 

Our previous studies on in situ intercalation polymerization have achieved the intercalation of several kinds of monomers into the interlayers of graphite, and the subsequent interlayer polymerization resulted in separating the graphite and carrying out in situ hybridization between the graphene layers and the polymers [21,22,23,24,25]. First-principles simulations indicate that the intercalations of aniline, pyrrole, and caprolactam are thermodynamics feasible [21,23]. Moreover, cationic monomers such as aniline cations (ANI^+^) can more easily achieve intercalation [21]. These works offer a novel perspective on the structural and functional regulation of graphene materials. Although the intercalation of organic monomers is important, the details about this process are still vague. Here, the intercalation mechanism of ANI^+^ is systematically studied by in situ intercalation polymerization designed with the response surface method (RSM), combined with molecular-dynamics (MD) and density functional theory (DFT)-based quantum chemical simulations. We expect this work to give a molecular-level perspective into the intercalation behaviors of organic monomers.

## 2. Materials and Methods

Materials: Natural graphite, namely, large flaky graphite (LfG), small flaky graphite (SfG), and microcrystalline graphite (MG), was purchased from Qingdao Yanhai Carbon Materials Co., Ltd. (Qingdao, China). Aniline, ammonium peroxydisulfate ((NH4)_2_S_2_O_8_, APS) and hydrochloric acid (HCl, 36 wt%) were purchased from Chengdu Kelong Chemical Co. Ltd. (Chengdu, China). Before using, aniline was distilled under reduced pressure (35 mmHg, 85 °C) and stored below 0 °C. APS powder and HCl solution were of analytical grade.

Preparation of PANI@GE hybrids. The PANI@GE hybrids were synthesized by in situ intercalation polymerization as in our previous reports [21,24]. In brief, 0.6 mL distilled aniline was dripped into 60 mL of HCl aqueous solution (1 M) to obtain the ANI^+^ solution. Then, 6 mg natural graphite was added into the solution, followed by ultrasonication to form a suspension. The species of graphite and the details of ultrasonication applied in each experiment were established using RSM [26] (see Table S1). Subsequently, the APS solution was prepared by dissolving 1.37 g APS into 30 mL HCl aqueous solution (1 M) and was dripped into the as-mentioned suspension with continuous stirring. The mixture was maintained for polymerization for up to 18 h. All the processes were performed in an ice bath (about 0−5 °C). The suspension was then filtered, and the precipitate was washed with ethanol and deionized water. After drying in an oven, the final product was ground into powder for further characterization.

Characterization. The morphologies of natural graphite were characterized with field-emission SEM (JSM-7001F, JEOL, Akishima, Japan). XRD patterns were tested on an X-ray diffractometer (Philips X’Pert PRO, PANalytical B.V., Netherlands) with Cu–Kα radiation. The compositions and electron-binding energy of samples were characterized by X-ray photoelectron spectroscopy (XPS, ESCALAB 250Xi, Thermo, Waltham, MA, USA).

Quantum chemical simulations. The geometry optimizations of aniline molecule (ANIm) and ANI^+^ were implemented with Gaussian 16 software [27] using a b3lyp exchange-correlation functional in conjunction with a 6–11g** basis set. The restrained electrostatic potential (RESP) of molecules was performed with Multiwfn 3.6 code [28]. Isosurface maps of their real space functions were rendered with Visual Molecular Dynamics (VMD) software [29].

Molecular dynamics simulations. MD simulations were executed using GROMACS 2018.3 [30]. A GROMOS 54A7 force field [31] was used for the MD simulations, and water molecules were simulated using the standard SPC/E model [32]. The force-field parameters of ANIm and ANI^+^ originated from Automated Topology Builder (ATB, https://atb.uq.edu.au/, accessed on 19 May 2021). To improve the accuracy, their atomic charges were replaced by the calculated RESP. The simulated models were constructed with VMD software and Packmol. The details of these models are described in Table 1. Energy minimization was first performed for all models. Then, the temperature of these systems was gradually increased from 0 K to the targeted 273.15 K within 100 ps, followed by an equilibrium process for 50 ps. Lastly, the systems ran for 10 ns at 273.15 K and 1 bar. Considering the constrained vibration of H atoms, the step size of MD simulations was 2 fs. The neighbor lists were generated with the default Verlet method. The particle-mesh Ewald (PME) and cut-off methods were applied to electrostatic and vdW interactions, respectively. Both of their cut-off distances were set to be 1 nm. The ultrasonication was simulated by giving a specific velocity to ANI^+^ according to Table S2. The step size of the intercalation process was reduced to prevent the collapse of systems induced by the fast-moving molecules.

First-principles calculations. Geometry optimizations and the electronic density difference of the adsorption models were calculated using CASTEP code [33] on the basis of DFT and plane-wave theory with the use of ultrasoft pseudopotentials. The generalized gradient approximation (GGA) of Perdew Burke and Ernzerhof (PBE) [34], and the dispersion-corrected DFT (DFT-D) [35], as implemented in the package, were employed in the simulation. The size of the periodic supercell for the simulation was 12.30 × 12.30 × 23.40 Å. The k-point grids were 2 × 2 × 1, and the cut-off energy for the plane-wave basis set was 400 eV.

## 3. Results and Discussion

In situ intercalation polymerization involves the intercalation of monomers and confined space polymerization [21,24]. As a prerequisite, the intercalation process plays a key role in the exfoliation and simultaneous in situ hybridization of graphite layers. Cationic monomers are easier to be drawn into the interlayer of graphite [21,23]. Therefore, aniline cations were chosen for intercalation with the use of ultrasonication. The intercalation of APS seems to not be an essential factor in intercalation polymerization. Previous works proved that concentrated sulfuric acid (98 wt%) is necessary for achieving the cointercalation of APS into graphite interlayers [36,37]. In this process, water should be strictly avoided or the rapid deintercalation of APS occurs. In this work, a diluted acid solution was used for dissolving aniline and APS, which goes against the intercalation of APS. Therefore, this work majorly focuses on the intercalation process of aniline cations. To investigate the influence of intercalation process on the as-prepared polyaniline@graphene (PANI@GE) hybrids, the response surface method was applied for the experimental design. The power and time of ultrasonication are denoted as X1 and X2, respectively. X3 refers to the species of graphite, namely, LfG, SfG and MG. Detailed experimental designs are listed in Table S1, and the obtained PANI@GE hybrids were characterized by XRD. Taking Experiment 9 as an example, the XRD pattern of the synthesized hybrids is presented in Figure S1. The diffraction peak was deconvoluted into eight peaks. The peak located at 2θ = 26.4° was attributed to the unexfoliated graphite in hybrids [21], and the other peaks were derived from the aligned PANI or dopant molecules [38]. The XPS spectra of PANI@GE hybrids are shown in Figure S2. The deconvoluted N 1s were in accordance with our previously reported results, indicating the aniline polymerizes between graphite interlayers [24]. Stacked graphite should be exfoliated during in situ intercalation polymerization, leading to the intensity decrease in the graphite (002) diffraction peak [24]. Obviously, the relative intensity ratio of graphite and polyaniline, recorded as Y, is available for evaluating the exfoliation of graphite. The calculated Y of each experiment is given in Figure S1.

**Figure 1:**
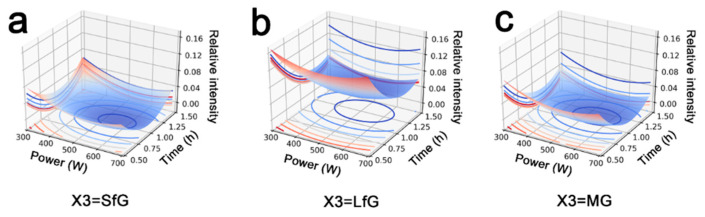
Effect of the exfoliation of graphene (Y) to ultrasonication power (X1) and time (X2) when the graphite is (**a**) small flaky graphite (X3 = SfG), (**b**) large flaky graphite (X3 = LfG), and (**c**) microcrystalline graphite (X3 = MG).

Linear regression analysis was performed according to the experimental parameters and results. A quadratic model was used considering the first-order (X1, X2, X3) and second-order (X1 · X1, X2 · X2, X3 · X3) influences of each term with their interaction terms (X1 · X2, X1 · X3, X2 · X3). The general-purpose expression of this model is presented as Equation (1), and the fitting regression coefficients are given in Table S3. Following the fitting results, relationships between graphite exfoliation and intercalation parameters are given in Figure 1.

(1) $$R=a+\mathrm{bX}1+\mathrm{cX}2+\mathrm{dMatch}\left(X3\right)+\mathrm{eX}1\;\cdot \;X2+\mathrm{fX}1\;\cdot \;\mathrm{Match}\left(X3\right)+\mathrm{gX}2\;\cdot \;\mathrm{Match}\left(X3\right)+\mathrm{hX}1\;\cdot \;X1+\mathrm{iX}2\;\cdot \;X2+\mathrm{jMatch}\left(X3\right)\;\cdot \;\mathrm{Match}\left(X3\right)$$

Overall, the synthesized PANI@GE hybrids exhibited lower Y values, as SfG or MG are used for in situ intercalation polymerization; thus, a better exfoliation degree of graphite is achieved. This phenomenon was majorly due to the structural differences between natural graphite. The SEM images and XRD patterns of these three kinds of graphite are given in Figures S3 and S4. The lamellar size of LfG was obviously larger than that of the others, and its graphite (002) diffraction peak also emerged at a higher position with a smaller full width at half maxima (FWHM). This evidence demonstrates its highly crystalline structure involving large and compactly stacked graphene layers, which hinders the intercalation of ANI^+^ and the swelling of graphite. Therefore, graphene layers in LfG are fairly difficult to be exfoliated as compared to SfG and MG.

The above experimental results indicate that ultrasonication significantly influences the exfoliation of graphite during in situ intercalation polymerization. Although ultrasonication is widely used for promoting the intercalation of monomers [21,22,23,39], its improving mechanism is still unclear. The dynamic behaviors of ANI^+^ intercalating into graphite interlayer are fuzzy. To reveal the dynamic process of the intercalation stage, MD simulations were applied together with DFT-based quantum chemical simulations.

**Figure 2:**
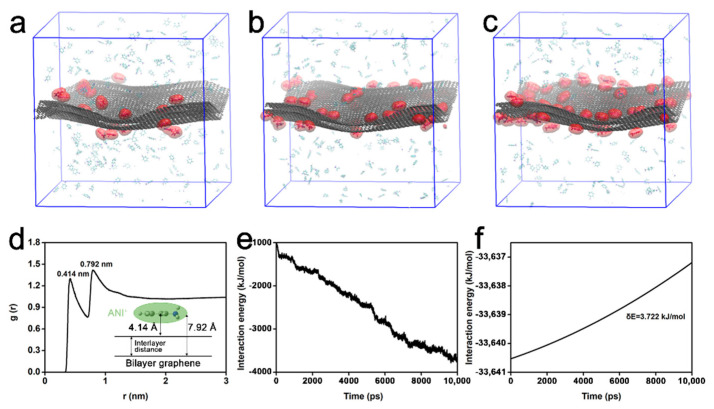
MD simulations of ANI^+^ adsorbed on the surface of bilayer graphene. (**a**–**c**) Snapshots of the adsorption process with simulated times of (**a**) 0 ns, (**b**) 5 ns, and (**c**) 10 ns. ANI^+^ within 0.5 nm from graphene is presented using the van der Waals surface model (red), as the others were the transparent line model. H_2_O and Cl^−^ are hidden to facilitate observation. (**d**) Radial distribution function between the centroid of ANI^+^ and carbon atoms in bilayer graphene, and (insert) their adsorption model. (**e**,**f**) van der Waals interaction energy between (**e**) ANI^+^ and bilayer graphene, and (**f**) graphene layers in bilayer graphene.

Once graphite had been added into the ANI^+^ solution, most of ANI^+^ moved to the surface of graphite because of its large surface area. Figure 2 is the MD simulation assuming the graphite having been immersed into ANI^+^ solution. To simplify the model, a piece of bilayer graphene was used for simulating the graphite block. The complete animation is provided in Video S1. Figure 2a–c present the snapshots with simulated times of 0, 5, and 10 ns, respectively, clearly demonstrating the gradual adsorption of ANI^+^ onto the surface of graphene. The stable adsorbing structure was further investigated by calculating the radial distribution function (RDF) between ANI^+^ and graphene. The curve in Figure 2d exhibits two individual peaks at r = 4.14 Å and 7.92 Å. The former indicates the average adsorption height of the lying ANI^+^. The latter peak indicates the average distance between ANI^+^ and another graphene layer. The adsorption model is described in the insert of Figure 2d. Thus, the average interlayer distance between the graphene layers was calculated to be 3.78 Å. Generally speaking, the interlayer distance between the graphene layers was 3.34 Å due to π–π interactions. The enlarged interlayer distance in the simulated results was reasonably attributed to the adsorption of ANI^+^.

On the basis of MD simulations, the van der Waals (vdW) interaction energy was extracted to explain the enlargement of the interlayer distance in bilayer graphene. Figure 2e provides the vdW interaction energy between ANI^+^ and bilayer graphene. The decrease in interaction energy was majorly due to the gradual adsorption of ANI^+^, which further weakened the π–π interactions between graphene layers, as proved by the increase in interlayer vdW interaction energy (Figure 2f). The adsorption of ANI^+^ was still unsaturated until 10 ns, as proven by the ceaselessly decrease in vdW interaction energy. Thus, the interlayer interactions between graphene layers are further weakened if the dynamic conditions (including time and temperature) permit it.

**Figure 3:**
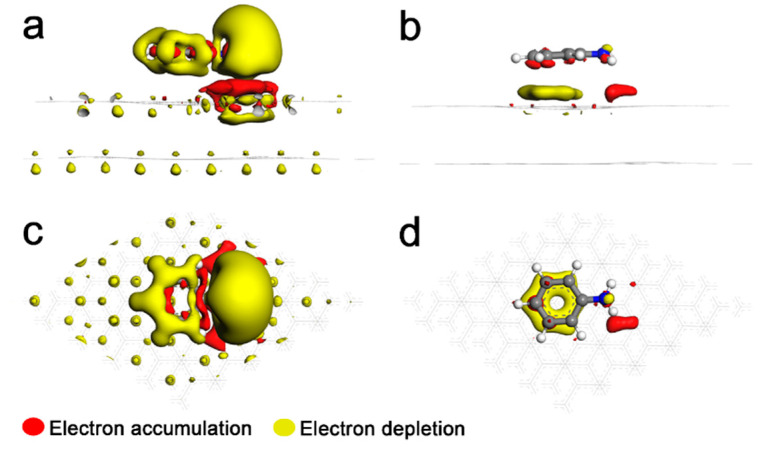
First-principles simulations of the adsorption models. Electronic density difference between bilayer graphene and (**a**,**c**) ANI^+^, and (**b**,**d**) ANIm with an isovalue of 0.003.

**Figure 4:**
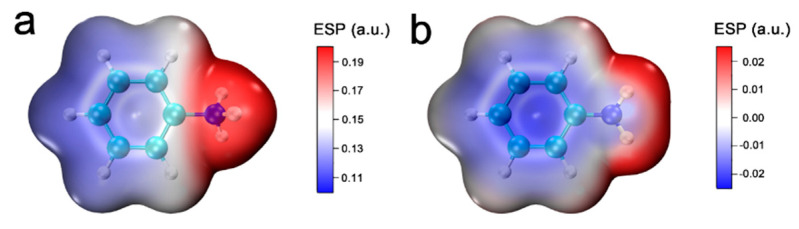
RESP-mapped van der Waals surface. (**a**) ANI^+^, (**b**) ANIm.

The MD simulation confirmed the interactions between the adsorbed ANI^+^ and graphene, but their interaction mechanisms are still unclear. Therefore, a model including ANI^+^ adsorbing on bilayer graphene was simulated with first-principles calculation. Their electronic density differences were calculated as shown in Figure 3a,c. It is clear that a large-scale transfer of the electronic cloud existed between ANI^+^ and graphene. Both of their electronic clouds accumulated on the upper surface of bilayer graphene. Moreover, the interlayer electronic clouds of bilayer graphene were intensely influenced, tending to transfer to the positive area (–NH_3_^+^) in the system. As a comparison, the adsorption model involving aniline molecule is provided in Figure 3b,d. Obviously, the electronic clouds transfer on a relative small scale and majorly increase near the benzene ring of ANIm. To explore their intrinsic interactions, RESP-painted vdW surfaces (namely, the isosurface of *ρ* = 0.001 a.u.) were rendered as shown in Figure 4. Figure 4b shows that ANIm was electronegative around its benzene ring due to its intrinsic aromaticity. Therefore, the interactions between ANIm and graphene are majorly described as π–π stacking. However, the cationic azyl conspicuously affected the electrostatic potential of ANI^+^. Both –NH_3_^+^ and the benzene ring were strongly electropositive (Figure 4a), indicating the forfeiting of aromaticity. Therefore, ANI^+^ was adsorbed on the graphene layers by the stronger electrostatic force rather than π–π interactions.

**Figure 5:**
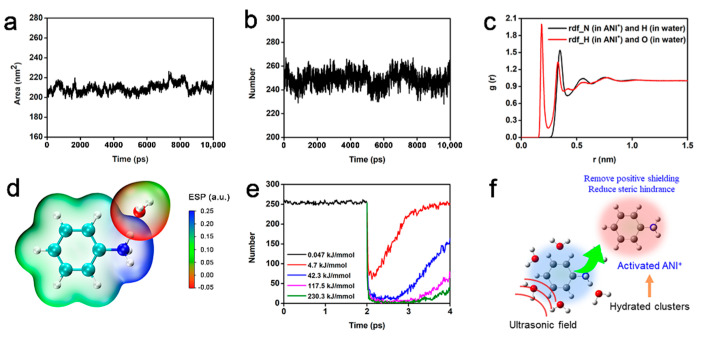
Interactions between ANI^+^ and H_2_O in solution. (**a**) Solvent-accessible surface area of ANI^+^ in solution; (**b**) number of hydrogen bonds formed between ANI^+^ and H_2_O; (**c**) radial distribution function between ANI^+^ and H_2_O; (**d**) RESP mapped van der Waals surface of ANI^+^ and H_2_O; (**e**) number of hydrogen bonds between ANI^+^ and H_2_O influenced by simulated ultrasonication; (**f**) schematics of the activated ANI^+^.

The adsorption of ANI^+^ weakened the interlayer interactions of graphite and enlarged its interlayer distance, facilitating the intercalation of other ANI^+^. However, another intercalation simulation manifested little ANI^+^ intercalating into the interlayer graphite within 10 ns (see Figure S5), as recorded in Video S2. According to previous works, the intercalation of ANI^+^ into graphite is thermodynamically feasible [21]. Consequently, the reason of the unsuccessful intercalation seems unclear. To find the truth, it is necessary to understand the structural characteristics and dynamic behaviors of the ANI^+^ solution in a free state. The following MD simulation of the ANI^+^ solution involved 100 ANI^+^ and 100 Cl^−^. The solvent-accessible surface area (SASA) of ANI^+^ is shown in Figure 5a. SASA refers to the numbers of H_2_O molecules that could access aniline cations, which can evaluate the dissolution of ANI^+^ in an aqueous solution. The SASA for ANI^+^ was about 210 nm^2^ during the whole simulation, indicating the essence of a homogeneous solution. Figure 5b shows that the number of hydrogen bonds formed between ANI^+^ and H_2_O was about 250. These hydrogen bonds remained steady for 10 ns, implying the formation of stable hydrated clusters. Considering the 100 ANI^+^ in the solution, each ANI^+^ formed 2.5 hydrogen bonds with H_2_O. Generally, –NH_3_^+^ in ANI^+^ provides two possible kinds of hydrogen bond structure, one of which forms between an N atom in ANI^+^ and an H atom in H_2_O, and the other is between an H atom in the –NH_3_^+^ of ANI^+^ and an O atom in H_2_O. RDF were calculated to confirm the actual structure of the hydrogen bonds. The black line in Figure 5c with a peak located at r = 0.35 nm indicates the vdW interaction-induced adsorption between N in ANI^+^ and H in H_2_O. However, the red curve exhibits another sharp peak at r = 0.18 nm, probably because of the strong hydrogen bond recorded as N^+^–H–O. The interpenetrating vdW surface presented in Figure 5d further proves the electrostatic force-driven interactions between the electropositive H in –NH_3_^+^ and electronegative O in H_2_O—in other words, the predicted N^+^–H–O hydrogen bond.

The hydrated ANI^+^ clusters partly shielded the positive charge of –NH_3_^+^ because of the hydrogen bond-bound H_2_O, which meant the weakening of the electrostatic interactions between ANI^+^ and the interlayer electronic clouds of graphite. On the other hand, clusters rather than a single ANI^+^ resulted in large steric hindrance during intercalation into the graphite. Thus, the hydrated clusters seem to be a major hindrance of the intercalation process. The following equation was used to evaluate the strength of the N^+^–H–O hydrogen bonds in the hydrated ANI^+^ clusters. The obtained formation energy E_binding_ = 0.94 eV of each hydrogen bonds indicates the spontaneous formation of clusters in the solution. Considering the number of hydrogen bonds formed by each ANI^+^, the formation energy of clusters in solution was about 0.23 kJ/mmol.

(2) $$E_{\mathrm{binding}}=E_{{\mathrm{ANI}}^{+}}+E_{H_{2}O}-E_{{\mathrm{ANI}}^{+}/H_{2}O}$$

It was experimentally proved that ultrasonication applied during intercalation is effective in improving efficiency of in situ intercalation polymerization. However, it was almost impossible to attach the ultrasonication field in MD simulations. From the perspective of molecules, ultrasonication exceedingly increases their instantaneous kinetic energy. Thus, specific velocities are given to ANI^+^ to simulate the effects of ultrasonication. The relationships between the velocities and kinetic energy of ANI^+^ were calculated and are shown in Table S2. In a free state, the average kinetic energy of ANI^+^ in a solution was about 0.047 kJ/mmol, and about 250 hydrogen bonds existed in the system, as presented in Figure 5e. As soon as the simulated ultrasonication was given, the number of hydrogen bonds dramatic declined for a while. This result indicates that the ANI^+^ dissociated from the hydrated clusters with the assistance of ultrasonication. The individual ANI^+^ in solution not only recovered the shielded positive charge, but also decreased its steric hindrance during intercalation.

**Figure 6:**
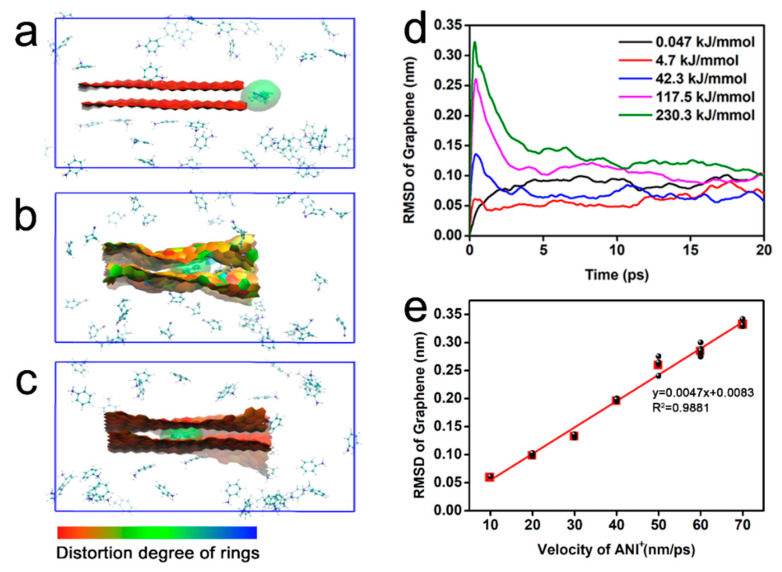
MD simulations of ANI^+^ intercalating into the interlayer of bilayer graphene. (**a**–**c**) Snapshots of the aniline cations intercalating into the interlayer of bilayer graphene with a simulated time of (**a**) 0 ps, (**b**) 0.3 ps, and (**c**) 3 ps. Water molecules are hidden in the snapshots. The graphene is presented using paper chain model to exhibit its distortion. The intercalating aniline cations are presented using a van der Waals surface model (green). (**d**) RMSD curves of bilayer graphene intercalated by aniline cations with different initial kinetic energy; (**e**) relationships between the maximal RMSD values and the initial kinetic of aniline cations.

Moreover, ultrasonication may lead to the differential intercalation behavior of ANI^+^. As presented in Figure 6a–c and Video S3, the accelerated ANI^+^ rapidly penetrated the interlayers within 0.3 ps and brought about the conspicuous deformation of graphene layers. The root mean square difference (RMSD) of graphene was calculated to measure the structural deviation from its initial state. Although the initial kinetic energy of ANI^+^ was different, all RMSD curves exhibited peaks at around 0.3 ps, as shown in Figure 6d, indicating the maximal deformation of graphene. Obviously, the accelerated ANI^+^ overcame the interlayer interactions during intercalation. In this process, the kinetic energy of ANI^+^ was consumed and transformed into the potential energy of graphene. However, the extremely fast intercalation provided little time for the release of energy, momentarily forming a microcanonical ensemble. The unreleased potential energy thus led to the dramatic deformation of the graphene layers. With more time, the stored energy was gradually released into the external environment by the movement of the layers. The RSMD consequently decreased to a stable value. Therefore, the RMSD of graphene seems to be available for evaluating the intercalation of ANI^+^. The maximal RMSD exhibited linear correlation to the initial velocity of ANI^+^, with a correlation coefficient of R^2^ = 0.9881 as depicted in Figure 6e. This phenomenon further confirmed the effectiveness of ultrasonication for promoting the intercalation of ANI^+^.

## 4. Conclusions

In situ intercalation polymerization is an effective method for the exfoliation and in situ hybridization of graphite layers, and the intercalation of monomers plays a key role in this process. On the basis of the above results, the intercalation mechanism of ANI^+^ was proposed here. Once graphite came into contact with an ANI^+^ solution, these cations were gradually adsorbed onto the surfaced of graphite. Although the adsorbed ANI^+^ significantly weakened the interlayer interactions in graphite, the determinant for intercalation seemed to be the ultrasonication applied in the intercalation process. The ultrasonic field broke up the hydrated clusters formed between ANI^+^ and H_2_O by strong hydrogen bonds. The positive charge of –NH_3_^+^ previously shielded by H_2_O consequently recovered, strengthening the electrostatic interactions between ANI^+^ and the interlayer π electronic clouds of graphite. The individual ANI^+^ also reduced its steric hindrance during intercalation. Therefore, the activated ANI^+^ could intercalate into graphite by consuming its initial kinetic energy provided by ultrasonication. This work demonstrated the dynamic intercalation behaviors of cations. The key factors for achieving intercalation were proposed with their influence mechanism, which paves the way for the structural design and functional regulation of organic-molecule-intercalated graphite compounds.

## Figures and Tables

**Table 1:** Descriptions of models for MD simulations.

Description	Components
Adsorption model	Cube box of 1.07 × 10.21 × 10.00 nm comprising a piece of periodic bilayer graphene, 200 ANI^+^, 200 Cl^−^, and 29,609 H_2_O.
Intercalation model	Cube box of 6.00 × 6.00 × 3.00 nm comprising a piece of nonperiodic bilayer graphene (4.91 × 3.26 × 0.34 nm), 40 ANI^+^, 40Cl^−^, and 2697 H_2_O.
ANI^+^ solution	Cube box of 5.00 × 5.00 × 5.00 nm comprising 100 ANI^+^, 100 Cl^−^, and 3369 H_2_O.

## Data Availability

The data is available on reasonable request from the corresponding author.

## Supplementary Materials

The following supporting information can be downloaded at: https://www.mdpi.com/article/10.3390/nano12142486/s1. Table S1: experimental design using RSM and corresponding results for the in situ intercalation polymerization. Table S2: kinetic energy of ANI^+^ calculated from their average velocity. Figure S1: Deconvolution of the XRD pattern of PANI@GE hybrids synthesized by in situ intercalation polymerization. Table S3: regression analysis for the in situ intercalation polymerization models of processing factors (X1, X2, X3) and the exfoliation degree of graphene (Y). Figure S2: SEM images of the natural graphite. Figure S3: XRD patterns of natural graphite. Figure S4: snapshots of few aniline cations achieving the intercalation simulated by molecular dynamics. Figure S5: Snapshots of few aniline cations achieving the intercalation simulated by molecule dy-namic with a simulated time of (a) 0 ns, (b) 5 ns and (c) 10 ns. Video S1: ANI cations gradually absorb on the surface of bilayer graphene (total 10 ns, every 20 ps per frame). Video S2: Few ANI cation achieves the intercalation(total 10 ns, every 20 ps per frame). Video S3: ANI cations successfully intercalate into the graphite with the assistance of ultrasonication (total 3 ps, every 10 fs per frame).
