# Supplementary material for: Mechanism for the Intercalation of Aniline Cations into the Interlayers of Graphite
Source: Nanomaterials (Basel). 2022 Jul 20;12(14):2486. doi: 10.3390/nano12142486 (PMC9318139; doi:10.3390/nano12142486)
Supplement: Supplementary file 1 [file nanomaterials-12-02486-s001.zip › nanomaterials-1777894 - Supporting Information.pdf]

Supporting Information

Mechanism for the Intercalation of Aniline Cations into the Interlayers of Graphite

Yifan Guo [1,2,3], Ying Li [4], Wei Wei [1,3], Junhua Su [1], Jinyang Li [1,3,*], Yanlei Shang [1], Yong Wang [1], Xiaoling Xu [1,3], David Hui [5] and Zuowan Zhou [1,2,3,*]

[1] Key Laboratory of Advanced Technologies of Materials (Ministry of Education), School of Materials Science and Engineering, Southwest Jiaotong University, Chengdu 610031, China; yfguo@swjtu.edu.cn (Y.G.); tzweir@swjtu.edu.cn (W.W.); sjh@my.swjtu.edu.cn (J.S.); ylshang@my.swjtu.edu.cn (Y.S.); yongwang1976@163.com (Y.W.); bihan_2001@163.com (X.X.)
[2] Research Institute of Frontier Science, Southwest Jiaotong University, Chengdu 610031, China
[3] Yibin Research Institute, Southwest Jiaotong University, Yibin 644000, China
[4] School of Mechanical Engineering, Chengdu University, Chengdu 610106, China; liying@cdu.edu.cn
[5] Department of Mechanical Engineering, University of New Orleans, New Orleans, LA 70148, USA; dhui@uno.edu
* Correspondence: jinyang.li@swjtu.edu.cn (J.L.); zwzhou@swjtu.edu.cn (Z.Z.)

Table S1. Experimental design using RSM and corresponding results for the *in situ* intercalation polymerization.

NO.	Ultrasonication		Graphite X3[a]	Exfoliation degree Y [b]
	Power X1(W)	Time X2 (h)		
**1**	**300**	0.5	SfG	0.08611
2	500	0.5	SfG	0.03967
3	700	0.5	SfG	0.04294
4	300	1.5	SfG	0.05965
5	700	1.5	SfG	0.04536
6	300	1.0	MG	0.01974
7	500	1.5	MG	0.00685
8	300	0.5	LfG	0.16364
9	700	0.5	LfG	0.16800
10	500	1.0	LfG	0.04263
11	300	1.5	LfG	0.07983
12	700	1.5	LfG	0.05856

[a] X3 refers to the species of graphite, which is regarded as a discrete variable during experimental design and data fitting. SfG, LfG and MG refers to the small-flaky graphite, large-flaky graphite and microcrystalline graphite respectively.
[b] Y are calculated by the relative intensity of graphite and polyaniline in the XRD pattern of PANI@GE hybrids.

Table S2. The kinetic energy of ANI^+ calculated from their average velocity.

Velocity of ANI^+ ($nm \cdot ps^{-1}$)	Kinetic energy of ANI^+ ($kJ \cdot mmol^{-1}$)
1	0.047
10	4.7
20	18.8
30	42.3
40	75.2
50	117.5
60	169.2
70	230.3

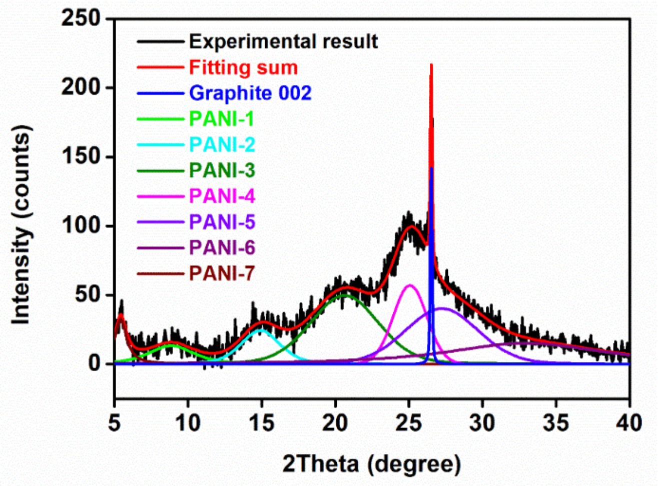

Figure S1. Deconvolution of the XRD pattern of PANI@GE hybrids synthesized by *in situ* intercalation polymerization.

Table S3. The regression analysis for the *in situ* intercalation polymerization models of the processing factors (X1, X2, X3) and the exfoliation degree of graphene (Y).

X3	=SfG	=LfG	=MG
		R^2=0.986933	
a		0.43975737204167	
b		-0.0006746918396	
c		-0.4564358215	
d	-0.0507283199167	0.06751952783334	-0.0167912079167
e		0.000004056815	
f	-0.0000227141667	0.00002797921083	-0.0000052650442
g	0.0423027525	-0.0423027525	0
h		0.0000006215264	
i		0.20004190699999	
j		0	

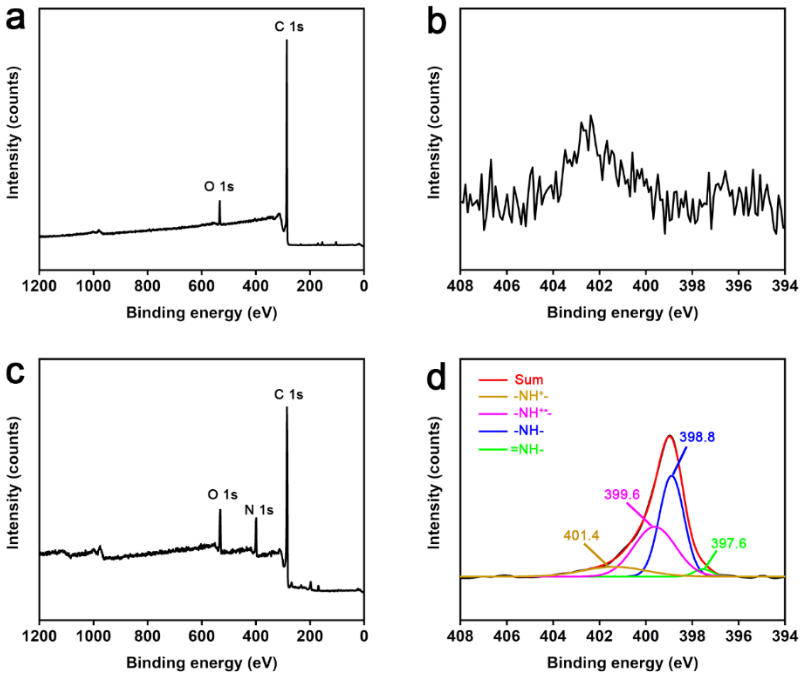

Figure S2. XPS spectra of natural graphite and PANI@GE. (a, b) XPS survey and high-resolution N 1s spectra of nature graphite. (c, d) XPS survey and high-resolution N 1s spectra of PANI@GE. The high-resolution spectrum is deconvoluted into 4 peaks.

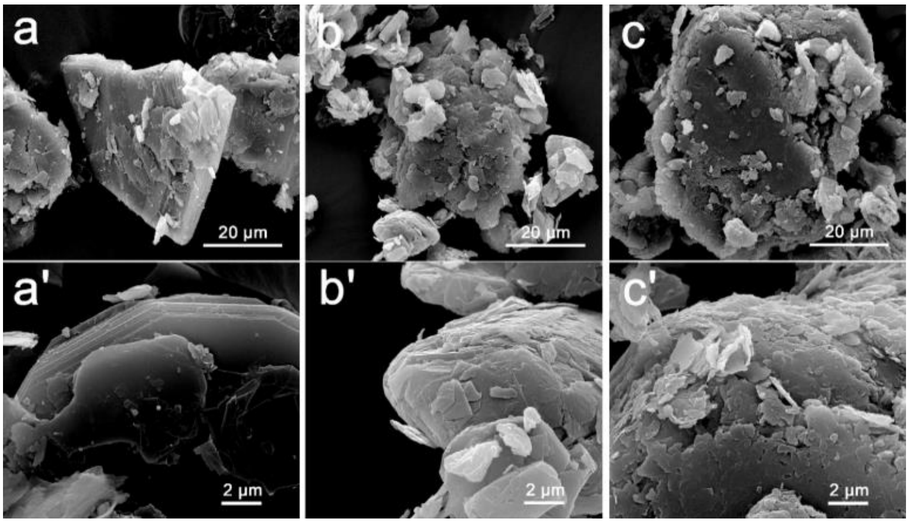

Figure S3. SEM images of the natural graphite. (a, a') Large-flaky graphite, (b, b') small-flaky graphite and (c, c') microcrystalline graphite.

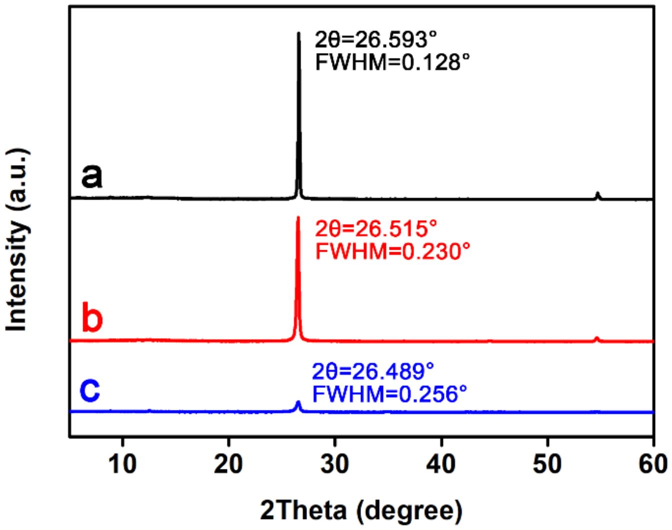

Figure S4. XRD patterns of natural graphite. (a) Large-flaky graphite, (b) small-flaky graphite and (c) microcrystalline graphite.

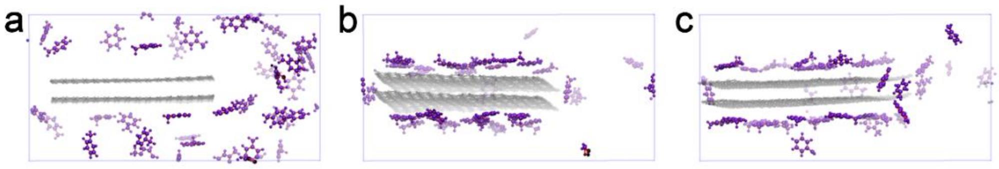

Figure S5. Snapshots of few aniline cations achieving the intercalation simulated by molecule dynamic with a simulated time of (a) 0 ns, (b) 5 ns and (c) 10 ns.
